# Assessment of the Use of Natural Materials for the Remediation of Cadmium Soil Contamination

**DOI:** 10.1371/journal.pone.0157547

**Published:** 2016-06-24

**Authors:** Tatiana de O. Pinto, Andrés C. García, Jair do N. Guedes, Nelson M. B. do A. Sobrinho, Orlando C. H. Tavares, Ricardo L. L. Berbara

**Affiliations:** Soil Science Department, Federal Rural University of Rio de Janeiro (UFRRJ), Rodovia BR 465, Km 07, Seropédica-Rio de Janeiro, CEP, Brazil; NERC Centre for Ecology & Hydrology, UNITED KINGDOM

## Abstract

Rice plants accumulate cadmium (Cd^2+^) within the grain, increasing the danger of human exposure. Natural materials have been used in soil remediation, but few studies have examined the risks (based on the bioavailability of these metals to plants) of using these materials, so the practice remains controversial. In the present study, we evaluated the effectiveness of biochar produced from sugarcane bagasse, vermicompost (VC), vermicompost solid residue (VCR) and humin for remediation of Cd^2+^-contaminated soils. We characterized the interactions between these materials and Cd^2+^ and evaluated their capacity to alter Cd^2+^ availability to rice plants. Our results show that under the conditions in this study, biochar and humin were not effective for soil remediation. Although biochar had high Cd^2+^ retention, it was associated with high Cd^2+^ bioavailability and increased Cd^2+^ accumulation in rice plants. VC and VCR had high Cd^2+^ retention capacity as well as low Cd^2+^ availability to plants. These characteristics were especially notable for VCR, which was most effective for soil remediation. The results of our study demonstrate that in the tested materials, the bioavailability of Cd^2+^ to plants is related to their structural characteristics, which in turn determine their retention of Cd^2+^.

## Introduction

Remediation of heavy metal (HM)-contaminated soils using materials of natural origin and with low environmental impact has been a viable strategy over the last few years [1–3]. Cadmium (Cd^2+^) contamination is of particular concern because Cd^2+^ occurs in almost all environments and is quickly mobilized by human activities such as mining [4]. In plants, Cd^2+^ uptake occurs through the roots, where its accumulation is high; Cd^2+^ is then translocated through the xylem via the apoplast or the symplast to the rest of the plant [5]. Cd^2+^ accumulation in rice plants presents a risk to human health because the metal accumulates within the rice grain [6,7]. Rice accumulates large amounts of Cd^2+^ in its tissues [8]; therefore, it is the cereal that introduces the most Cd^2+^ into the human diet through ingestion [7]. In addition, rice’s genetic characteristics permit Cd^2+^ accumulation and transport from the roots to the shoots and grains [9,10].

Many studies have examined the use of materials such as biochar, vermicompost (VC) and humic fractions for soil remediation with the goal of decreasing the impact of plant Cd^2+^ accumulation [11–15]. Biochar has been widely used over the past few years as a soil amendment [16,17] that provides plant protection [18,19] and improves crop yields [20,21]. VCs are known to improve soil quality [22] and plant growth [23] and have been used for remediating HM-contaminated soils [24]. Studies have also assessed the use of humic substances (HS) for soil remediation due to their high structural diversity, which favors interaction with HM [25,26]. Specifically, humin has a high HM retention capacity [27] and acts as an indicator of the stability of humified organic matter within the soil [28,29]. Despite these studies, the risks of using biochar, VC and HS for remediation of Cd^2+^-contaminated soils are not clear, and comparative studies of Cd^2+^ retention, availability and subsequent assimilation by plants are needed. We tested the hypothesis that the structural characteristics of biochar, VC and humin determine their interaction with Cd^2+^ and that the nature of this interaction influences Cd^2+^ uptake by plants.

The goals of the present study were to characterize the nature of Cd^2+^ retention in biochar, VC, VC solid residue (VCR) and humin and to evaluate the effects of these materials on the availability of Cd^2+^ to rice plants. The Langmuir and Freundlich adsorption isotherms were determined, and the materials were characterized before and after Cd^2+^ retention using cross-polarization/magic-angle spinning nuclear magnetic resonance spectroscopy (CP/MAS ^13^C-NMR) and Fourier transform infrared spectroscopy (FTIR). Multivariate analysis of the resulting data was performed. To study the capacity of the tested materials to decrease Cd^2+^ availability for plants, rice plants were grown in the presence of the tested materials and the retained Cd^2+^ and the Cd^2+^ contents of the plant tissues were quantified.

## Materials and Methods

### Studied materials (VC, VCR, biochar and humins)

VC produced from plant debris and cow manure was supplied by the Agroecological Farm (Fazenda Agroecológica) of EMBRAPA Agroecology, Seropédica, Brazil. VCR was obtained through HS extraction of the VC, following the methods of Garcia et al. [30]. Biochar was obtained from sugarcane bagasse and as a byproduct of bio-oil production. To obtain the biochar, pyrolysis was carried out at 650°C for 1 h. Scanning electron microscopy shows that the resulting material had the morphological characteristics of biochar (S1 Fig). Humin was obtained from organic soil collected in Santa Cruz, Baixada Fluminense, Rio de Janeiro, Brazil.

### Adsorption isotherms modeling of Cd^2+^ retention by the tested materials

Adsorption isotherms for Cd^2+^ retention were constructed using data obtained in the following manner. Cadmium chloride (CdCl_2_) solutions with a final volume of 100 mL and containing various concentrations of Cd^2+^ (1 mg L^-1^, 5 mg L^-1^, 20 mg L^-1^, 50 mg L^-1^ and 100 mg L^-1^) were added to 20 g of each tested material; the solutions were stirred for 240 minutes for biochar (pH = 4.2), 360 minutes for VC (pH = 5.0), 150 minutes for humin (pH = 4.8) and 190 minutes for VCR (pH = 5.0). Following stirring, the suspensions were filtered and supernatant Cd^2+^ concentrations were determined by atomic absorption spectroscopy (VARIAN 55B Atomic Absorption Spectrometer).

The retention data were fitted to a linear Langmuir isotherm according to the following equation:

$$\frac{{\text{C}}_{\text{e}}}{{\text{Q}}_{\text{e}}}=\frac{1}{{\text{bQ}}_{\text{max}}}+\frac{{\text{C}}_{\text{e}}}{{\text{Q}}_{\text{max}}}$$

where Ce is the equilibrium concentration of the adsorbate (mg L^-1^), Qe is the amount of metal adsorbed per gram of the absorbent at equilibrium (mg g^-1^), b is the Langmuir absorption constant at a given temperature (L mg^-1^), and Qmax is the maximum adsorption capacity (mg g^-1^).

A linear Freundlich isotherm was used, according to the following equation:

$$\text{log}\left(\text{Q}\right)=\text{log}{\text{K}}_{\text{F}}+\frac{1}{\text{n}}\text{log}{\text{C}}_{\text{e}}$$

where KF (mg g^-1^) is the sorption capacity and n is the sorption intensity.

### Spectroscopic characterization of materials

Infrared spectra of the materials were obtained in the spectral range from 4000–400 cm^-1^ using FTIR (Thermo Scientific Nicolet 6700) with KBr plates (5 mg of each material + 200 mg KBr).

CP/MAS ^13^C-NMR was performed using a Bruker AVANCE II 400 MHz NMR spectrometer equipped with a 4 mm narrow MAS probe and operated at a ^13^C resonance frequency of 100.163 MHz. Spectrum acquisition and processing was performed using the Bruker Topspin 2.1 software. The free induction decay (FID) was zero filled to 4 k and multiplied by an exponential weighing function corresponding to a line broadening of 70 Hz.

### Influence of material structure on Cd^2+^ retention

Chemometric principal component analyses (PCAs) of the CP/MAS ^13^C-NMR spectra and FTIR data were performed for all materials using the Unscrambler® X 10.3 software (Camo Software AS Inc., Oslo, Norway). ^13^C-NMR and FTIR spectra of the materials with and without retained Cd^2+^ were imported into the software, and their areas were normalized. PCA analyses for each material were performed using the NIPALS algorithm with CROSS VALIDATION.

### Cd^2+^ bioavailability to rice plants

#### Plant material and experimental conditions

Cd^2+^ uptake by rice plants (*Oryza sativa* L.) was investigated for the Piauí rice variety. Plants were grown in a growth chamber under a 12 h light/12 h dark photoperiod at 28°C/24°C (day/night), 70% relative humidity, and a light intensity of 250 μmol m^−2^ s^−1^. Rice seeds were surface-sterilized with 2% sodium hypochlorite for 10 minutes and rinsed with distilled water. The seeds were placed in jars with gauze soaked in distilled water for germination. Four days after germination, the seedlings were transplanted into jars containing washed and autoclaved sand mixed with each of the tested Cd^2+^ enriched materials (170 mg Cd^2+^ g^-1^ material). The Cd^2+^ values were obtained from the adsorption isotherms (the retained Cd^2+^ values were confirmed through quantification by atomic absorption spectrometry). Substrates were prepared using ratios selected based on experimental evidence obtained in our laboratories or previously reported. Biochar was applied at 30 g kg^-1^, a concentration that has been observed to improve soil conditions [31,32]; VC was applied at 200 g kg^-1^, a concentration that has been observed to increase plant production and improve soil conditions [30]; VCR was applied at 90 g kg^-1^, a concentration observed to retain heavy metals [30]; and humin was applied at 20 g kg^-1^, a concentration that is consistent with its natural abundance [33,34]. Following planting, ¼ strength Hoagland solution [35] was added to the substrate on each of the first three days, and ½ strength solution was added daily for the remainder of the experiment (pH 5.8). A completely randomized experimental design was used for all experiments; the experimental design included five plants per pot and ten replicates per treatment. The experiment was run for 28 days after transplantation. All determinations were made from four harvests spaced seven days apart.

### Root parameters

Assessments of root parameters were performed using WinRhizo Arabidopsis 2012b and the data were analyzed using XLRhizo (Regent Instruments, Quebec, Canada Inc.). Four root parameters were quantified and analyzed: length (mm), surface area (mm^2^), average diameter (mm) and the number of roots. Root length (mm) and the number of roots were classified as very fine (0.5–1.5 mm), fine (1.5–3.5 mm) or thick (> 3.5 mm). Roots and shoots were subsequently placed in an oven and dried at 105°C until they reached a constant weight, after which the leaf and root dry weight was determined.

### Cd^2+^ content of plant tissues

The Cd^2+^ content of the rice plants was quantified by atomic absorption spectrometry using a VARIAN 55B atomic absorption spectrometer (Agilent Technologies, Headquarters 5301 Stevens Creek Blvd, Santa Clara, CA 95051, United States). The roots and leaves were ground and digested using a mixture of concentrated nitric acid and perchloric acid (HNO_3_/HClO_4_). Following digestion, the samples were added to 25 mL of distilled water and filtered for Cd^2+^ determination.

### Exchangeable Cd^2+^ content and bioavailability

At the end of the experiment, the bioavailable Cd^2+^ was extracted from the substrates using deionized H_2_O; the exchangeable Cd^2+^ was extracted using MgCl_2_ (0.05 mol L^-1^) [36]. Phytoextraction indices were calculated based on the values for Cd^2+^ adsorbed by plants and accumulated in plant tissues. The bioconcentration factor (BCF) was calculated using the following equation: BCF = Cd^2+^ (plant tissue)/ Cd^2+^ (soil) [37]. The plant’s capacity for translocating Cd^2+^ from the root to the shoot was determined using the translocation index (TI): TI = Cd^2+^ (shoot)/ Cd^2+^ (root)*100 [38].

## Results and Discussion

### Modeling of Cd^2+^ retention using the Langmuir and Freundlich isotherms

**Fig 1:**
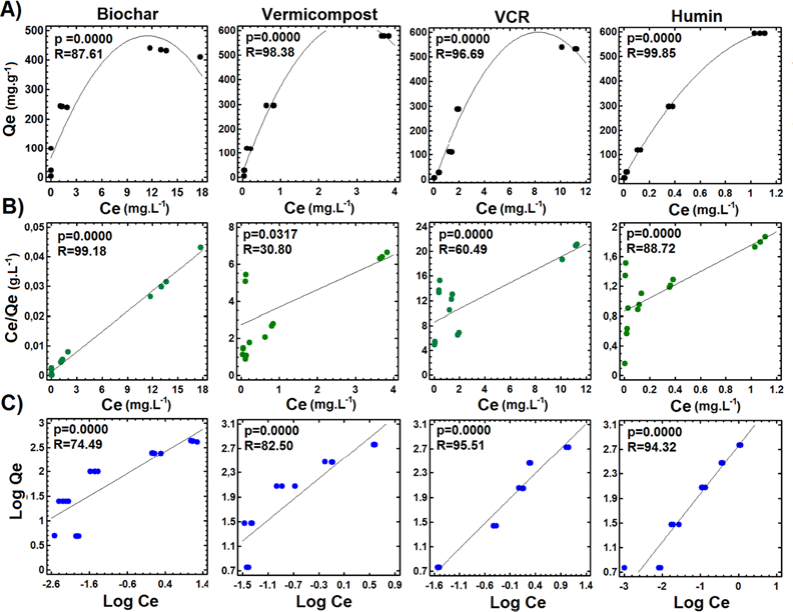
Cd^2+^ Adsorption Isotherms for the Tested Materials. From top to bottom: A) general adsorption isotherms, B) linear Langmuir isotherms and C) linear Freundlich isotherms. Retention values were determined through simple linear regression and were significant at p<0.05 (*n = 15*).

The four tested materials showed L-type adsorption isotherms, indicating Cd^2+^ interaction with the surface of the material until saturation of the available binding sites (Fig 1A) [39]. Linear Langmuir isotherms were the best-fitting isotherms for Cd^2+^ retention on biochar (R^2^ = 99.18, p<0.001), but these isotherms fit poorly for retention on VC (R^2^ = 30.80, p<0.001) (Fig 1B). Linear Freundlich isotherms were the best-fitting isotherms for Cd^2+^ retention on VCR (R^2^ = 95.51, p<0.001), humin (R^2^ = 94.32, p<0.001) and VC (R^2^ = 82.50, p<0.001) (Fig 1C).

The good fit of the Langmuir model for biochar indicates that retention occurred through monolayer formation, with retention sites presenting equivalent affinities for Cd^2+^ (Fig 1B) [40]. In contrast, the poor fit of the Langmuir model and the good fit of the Freundlich model for VC indicate that the binding sites had heterogeneous chemical characteristics. However, the good fit of the Freundlich model for VCR and humin indicates that these materials possess higher binding site diversity and heterogeneity than the other materials. Retention on these materials may initially result from chemical bonds and later from electrostatic interactions (Fig 1C) [40]

According to the adsorption parameters obtained from the Langmuir isotherms, VCR had the highest retention capacity, followed by biochar, VC and humin. VC and VCR presented lower b values than the other tested materials. The parameter b is related to the adsorption energy, indicating stronger interactions with Cd^2+^; in our samples, these may have been chemical interactions [30,41]. The parameter Kf, which can be obtained from the Freundlich isotherms and is related to the sorption relative capacity [30,41,42], was highest for VC and VCR. The value of n, which in our samples ranged from 1 to 10, is related to sorption intensity [30,41,42]; it indicated high retention by the four tested materials and was greatest for biochar (**Table 1).**

**Table 1:** Adsorption parameters obtained from Freundlich and Langmuir isotherms.

Materials	b (L mg^-1^)	Qmax (mg g^-1^)	Kf (mg g^-1^)	n
	Langmuir parameters		Freundlich parameters	
**VC**	0.34	206.8	283.85	1.18
**Biochar**	1.95	238.90	171.14	2.20
**VCR**	0.12	348.01	292.81	1.22
**Humin**	1.03	171.91	175.43	1.27

**Fig 2:**
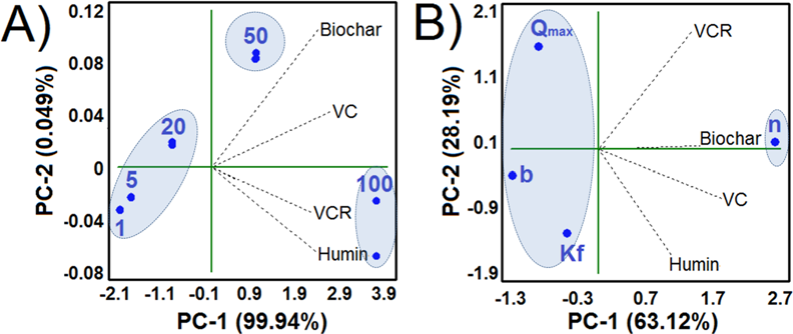
**PCA Showing the Relationship between (A) the Tested Materials (biochar, VC, VCR and humin) and Various Cd^2+^ Concentrations (1 to 100 mg Cd^2+^ L^-1^, in blue) and (B) the Tested Materials (biochar, VC, VCR and Humin) and the Langmuir and Freundlich Isotherm Parameters (Qmax, b, Kf and n, in Blue)**.

In the PCA, PC1 was observed to explain 99.9% of the total variance and showed that Cd^2+^ retention in the tested materials had a relationship with higher Cd^2+^ concentrations (Fig 2A). This result indicates that the differences in retained Cd^2+^ were more pronounced when the materials interacted with 50 and 100 mg L^-1^ Cd^2+^. The PCA for the adsorption isotherm parameters explained 91.31% of the total variance and showed a close relationship between the tested materials and n (PC1 63.12%) (Fig 2B), confirming favorable interactions between the materials and Cd^2+^.

### Characterization of Cd^2+^ retention

#### Characterization of retention using CP/MAS ^13^C-NMR

VC had a higher number of carboxyl groups (10.7%) than VCR (6.4%) and humin (7.1%); and VC and VCR had more aliphatic oxygenated and nitrogenous groups than humin (S1 Table). Biochar had a large number of substituted aromatic structures (Ar-O,N; 62.5%) and a relatively high number of carboxyl (8.6%) and carbonyl (2.8%) groups.

No differences in peak presence in the CP/MAS ^13^C-NMR spectra were observed among the four materials both with and without retained Cd^2+^ at any of the five Cd^2+^ concentrations tested (S2 Fig). In addition, no significant changes in the amounts of the structures detected through integration of spectral regions were observed, and there were no changes in aromaticity or aliphaticity due to Cd^2+^ retention (S1 Table).

**Fig 3:**
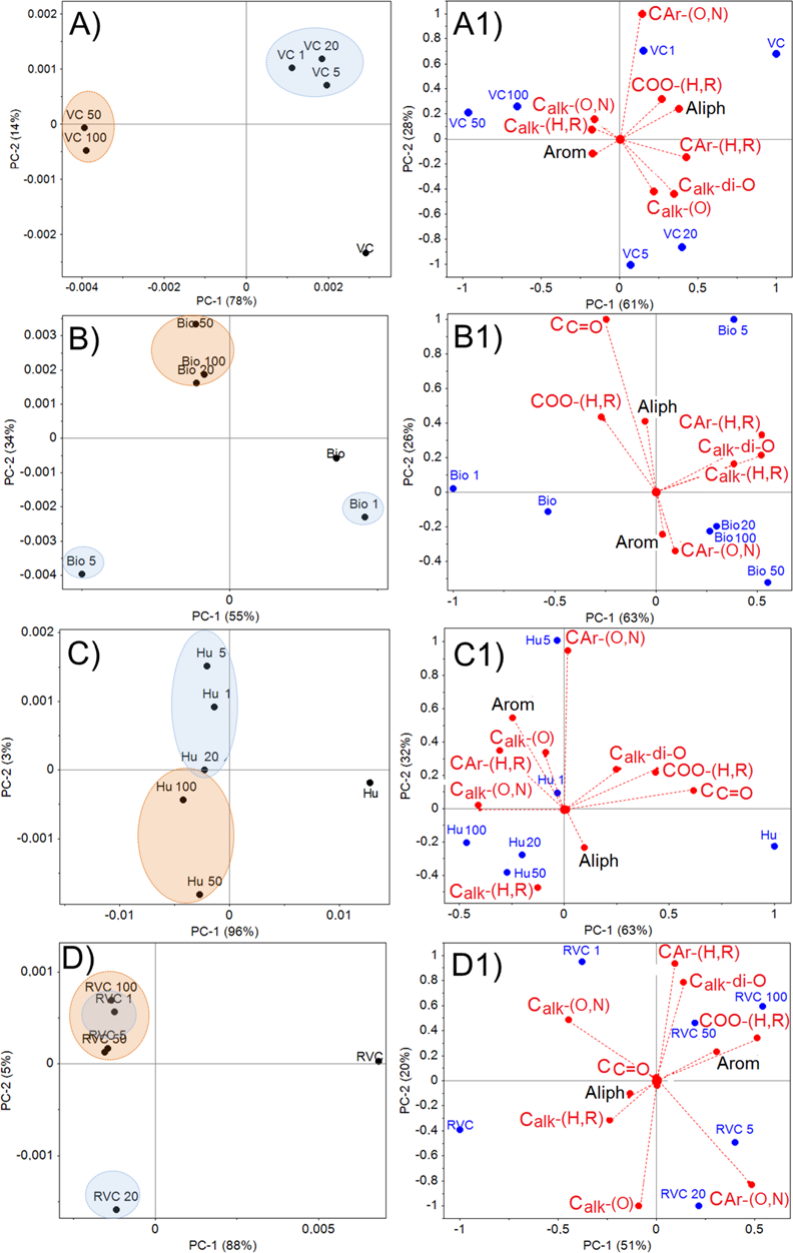
**PCA Analysis of CP/MAS ^13^C-NMR Pure Spectra (A, B, C, D and E) and the Carbon Type (A1, B1, C1, D1 and E1) of Materials with and without Various Levels of Retained Cd^2+^**.

PCA of the pure spectra (Fig 3A, 3B, 3C and 3D) revealed structural differences between the materials with and without retained Cd^2+^ at the highest Cd^2+^ concentrations tested. Concentrations of 50 and 100 mg L^-1^ Cd^2+^ resulted in the greatest structural differences for VC, indicated by their shifts to negative values for PC1 (78%) (Fig 3A). Structural changes in biochar were observed at Cd^2+^ concentrations greater than 5 mg L^-1^ (Fig 3B). Structural changes in humin and VCR were observed at the lowest Cd^2+^ concentration tested (1 mg L^-1^).

PCA of the spectra integrated by region showed that the different structures present in the tested materials had implications for the interaction of the materials with Cd^2+^ (Fig 3A1, 3B1, 3C1 and 3D1). Oxygenated moieties (-COOH, CAr-O, Calk-O and Calk-di-O) were related to VC with an without retained Cd^2+^ at concentrations of 1–20 mg L^-1^ but not at the highest Cd^2+^ concentrations (50–100 mg L^-1^). Thus, the interaction of VC with Cd^2+^ at high Cd^2+^ concentrations seems to result in changes to the more oxygenated side chains; these changes may result from chemical binding of Cd^2+^ (Fig 3A1). The interactions of biochar with Cd^2+^ at concentrations greater than 1 mg L^-1^ resulted in changes to the -COOH and -C = O groups (Fig 3B1). In contrast, in humin, all tested Cd^2+^ concentrations caused changes due to Cd^2+^ interactions with -COOH, C = O and Calk-di-O groups (Fig 3C1). In VCR, the Calk-O,N groups may act as available sites for Cd^2+^ retention at concentrations higher than 1 mg L^-1^ (Fig 3D1).

#### Characterization of retention through FTIR

The four tested materials had similar ionized functional groups, all of which favor interaction with Cd^2+^ (S3 Fig). Absorption bands at approximately 3,400 cm^-1^ were observed for all materials tested, consistent with stretching vibrations (ν) of–OH and/or–NH groups (alcohols, carboxylic acids and amides). Bands between approximately 1,620 cm^-1^ and 1,650 cm^-1^ indicate ν for C = C aromatic, C = O amide I, and symmetric–COO- groups, and those between approximately 1,035 cm^-1^ and 1,110 cm^-1^ belong to ν of–OH groups from aliphatic alcohols and polysaccharides (S3 Fig) [43–47].

**Fig 4:**
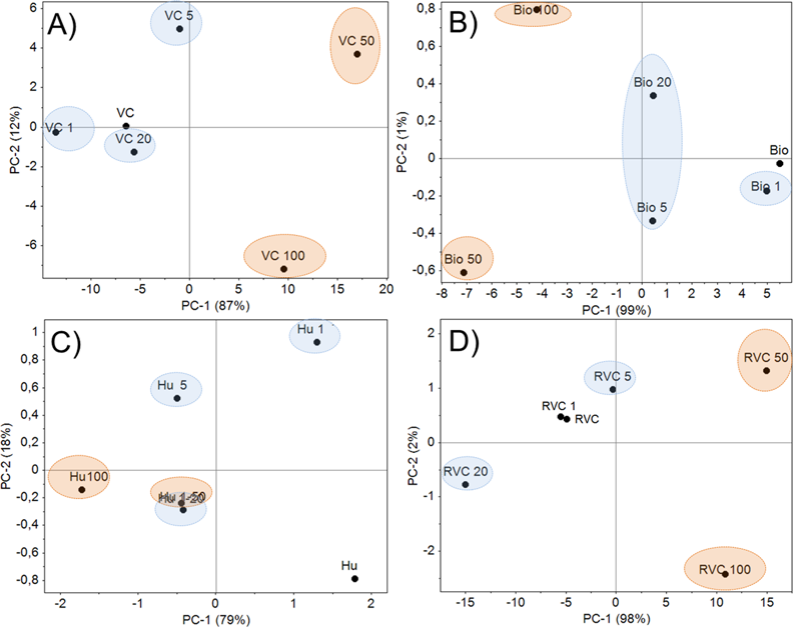
PCA of FTIR Pure Spectra of Materials with and without Various Levels of Retained Cd^2+^.

PCA was also performed on the spectra of materials with and without retained Cd^2+^ (Fig 4). This experiment revealed changes in functional groups that were not evident from visual analysis of the FTIR spectra. Differences in functional groups were observed in all materials tested at the highest Cd^2+^ concentrations and without Cd^2+^ (Fig 4). These results confirmed the PCA results for the ^13^C-NMR data.

Previous studies have confirmed the Cd^2+^ retention capacity of all tested materials except VCR (biochar, humin and VC) [48–53]. However, these studies examined the structural changes and chemical groups involved in Cd^2+^ retention using chemometric methods. We found that all tested materials had a high Cd^2+^ retention capacity (Table 1), with VCR and biochar displaying the highest amounts of retained Cd^2+^. Hydroxyl (-OH), -NH and -COO- groups (Fig 3), all of which are nitrogenated and oxygenated chemical groups (e.g., -COOH and -C = O) (Fig 3A1, 3B1 and 3C1), are directly involved in Cd^2+^ retention by biochar, VC and humin. Hydroxyl (–OH) and–NH groups in Calk-O,N structures may be the initial sites of Cd^2+^ retention in VCR (Fig 3, Fig 3D and 3D1).

### Cd^2+^ availability of the tested materials to rice plants

**Fig 5:**
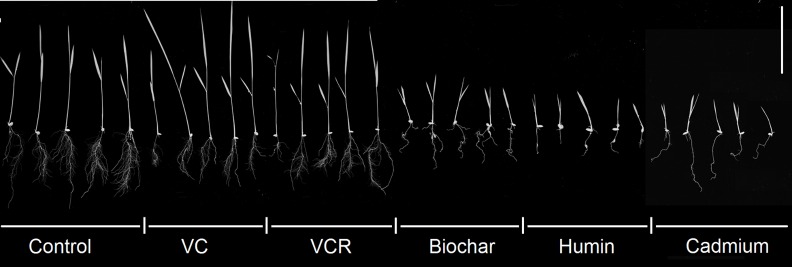
Sizes of Plants Grown on Substrates Containing Cd^2+^ 28 days after Seedling Transfer (DAT); bar is 50 mm.

The type of growth substrate affected plant growth. Growth inhibition was observed for plants grown in biochar and humin; this effect was similar to the toxicity produced by available Cd^2+^ (Fig 5).

**Fig 6:**
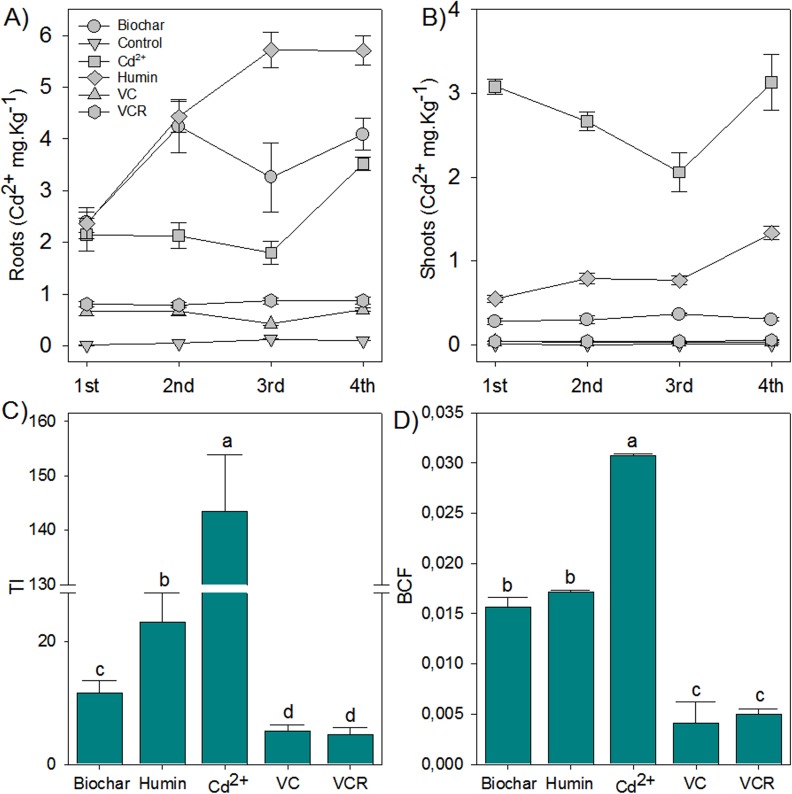
Root and Leaf Cd^2+^ Contents of Rice Plants from the First Harvest until 28 Days after Seedling Transfer (DAT) (A and B); on the x axis, 1st, 2nd, 3rd and 4th indicate, respectively, the first, second, third and fourth harvests from each experiment, conducted at seven-day intervals. Values are reported as averages ± the standard error (*n = 30*). (C) The translocation index (TI) and (D) the bioconcentration factor (BCF) were calculated at 28 DAT.

Quantification of Cd^2+^ in the roots and leaves of rice plants showed how much Cd^2+^ was released by each of the substrate materials (Fig 6A and 6B). Plants growing on substrates containing humin or biochar or with bioavailable Cd^2+^ had higher Cd^2+^ accumulation in roots. In contrast, plants growing in substrate containing VC and VCR had lower Cd^2+^ accumulation in roots, with slightly higher root accumulation than in the control plants. These results indicate that the Cd^2+^ retained in humin and biochar is readily available for plant uptake, whereas VC and VCR decrease the Cd^2+^ available for plant uptake. The plant Cd^2+^ contents suggested that Cd^2+^ is retained through weaker interactions in humin and biochar or that the Cd^2+^ binding sites in these materials are structurally more superficial than those in VC and VCR. Either of these conditions would give the plant roots better access to the metal; the interaction of these materials with roots, as well as acid exudation and resulting rhizosphere acidification, could facilitate the release of weakly or superficially retained Cd^2+^.

Plants grown in the presence of available Cd^2+^ exhibited greater Cd^2+^ translocation from roots to leaves (Fig 6B). Greater Cd^2+^ accumulation in leaves due to translocation was also observed for plants growing in substrates containing humin and biochar. This observation confirms that these materials increase the availability of Cd^2+^, allowing the Cd^2+^ to be accumulated by plants. In contrast, plants growing on VC and VCR had low Cd^2+^ translocation and little accumulation in leaves.The findings were confirmed by comparison of the translocation indices (TIs) of plants grown under different conditions (Fig 6C). The high TIs of plants grown in the presence of Cd^2+^ indicate that Cd^2+^ bioavailability strongly affects its accumulation in the shoot. Plants grown in humin and biochar containing Cd^2+^ had higher TIs than plants grown in VC and VCR, confirming the capacity of humin and biochar to increase the bioavailability of Cd^2+^. The Cd^2+^ bioconcentration factor (BCF) for plants grown with Cd^2+^ was also high due to this Cd^2+^ bioavailability (Fig 6D). Humin and biochar yielded the highest BCF values, again confirming the bioavailability of the Cd^2+^ in these substrates.

**Fig 7:**
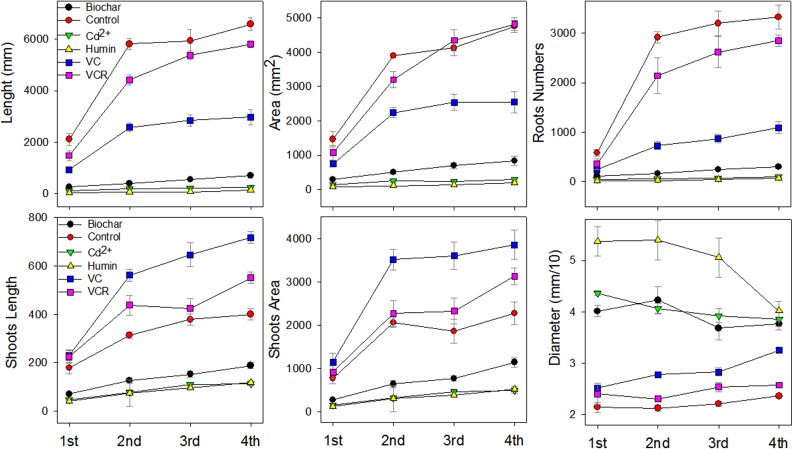
Root and Leaf Growth Parameters for Plants Grown in Different Substrates Containing Retained Cd^2+^ from the First Harvest until 28 Days after Seedling Transference (DAT); on the x axis, 1st, 2nd, 3rd and 4th, respectively, indicate the first, second, third and fourth harvests from each experiment, conducted at seven-day intervals. Values are reported as averages ± the standard error (*n = 30*).

Assimilation of Cd^2+^ resulted in varying levels of toxicity in plants grown in substrates containing the tested materials, and this assimilation affected plant growth (Fig 7). Compared with plants in the control treatment, root growth was inhibited in plants grown in all of the tested materials; the experimental plants had decreased root length, smaller root area and a decreased number of roots. These effects were less pronounced for plants grown in VC and VCR and more pronounced for plants grown in biochar and humin.

Root thickening was observed after plant growth in all materials containing Cd^2+^, confirming the detrimental effects of Cd^2+^. Increased root diameter (thickening) of the root may be a response to Cd^2+^ toxicity. Shoot growth did not present the same response as root growth for all tested materials. The leaf length and the area of plants grown in VC and VCR were higher relative to the control plants, and these parameters were lower for plants grown in humin and biochar relative to the control plants.

Biochar and humin release high amounts of Cd^2+^ during their interaction with roots (Fig 7). This bioavailable Cd^2+^ accumulates in roots and is translocated into the leaves, resulting in toxicity to the rice plants and inhibition of root and leaf growth and development (Fig 7). VC and VCR release lower amounts of bioavailable Cd^2+^ to plants than biochar and humins, resulting in lower Cd^2+^ accumulation in the roots and leaves relative to the other treatments. Thus, plant growth in VC and VCR produced lower Cd^2+^ toxicity, resulting in decreased root growth and increased shoot growth compared to the control (Fig 7)

**Fig 8:**
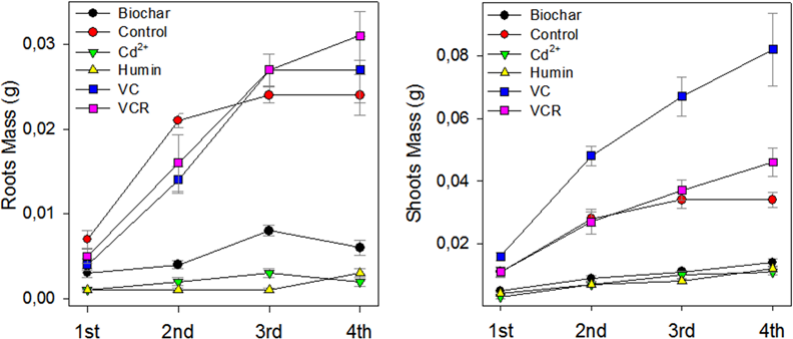
Root and Leaf Dry Weights of Plants Grown on Different Substrates Containing Retained Cd^2+^ from the First Harvest until 28 Days after Seedling Transfer (DAT); on the x axis, 1st, 2nd, 3rd and 4th, respectively, indicate the first, second, third and fourth harvests from each experiment, conducted at seven-day intervals. Values are reported as averages ± the standard error (*n = 30*).

Leaf and root biomass were also determined (Fig 8). A pronounced inhibitory effect on leaf and root biomass production was observed for plants grown with biochar and humin containing Cd^2+^, compared to the control and the other treatments. In contrast, plants grown with VC and VCR had similar root biomass as the control plants, and plants grown with VCR had higher leaf biomass than the control.

The effects on biomass production of growing plants in VC and VCR in the presence of Cd^2+^ may be attributed to an antioxidative defense response in which the synthesis of defense and transport proteins is increased in order to decrease Cd^2+^ toxicity [54–56]. Fragments of humic molecules present in humified materials may also have anti-stress effects through antioxidative protection [23,57] or may stimulate growth through hormone action [58,59].

**Fig 9:**
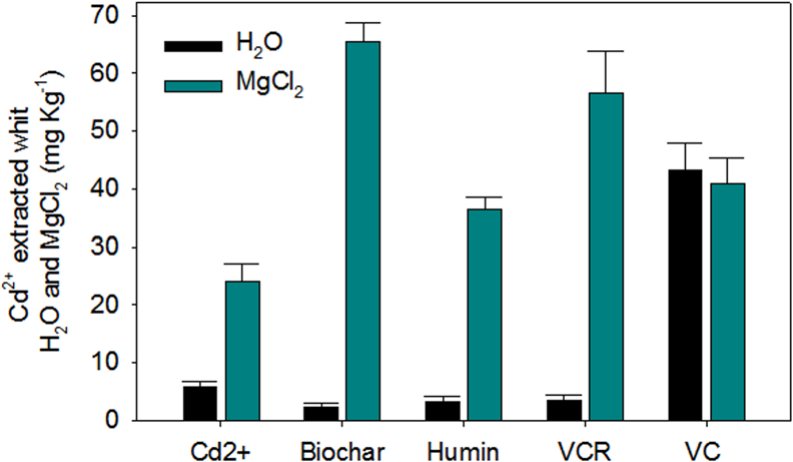
Amount of Bioavailable (H_2_O extraction) and Exchangeable (MgCl_2_ extraction) Cd^2+^ Extracted from the Substrates at the End of the Experiment (28 days after seedling transfer).

Quantification of the Cd^2+^ remaining in the substrates after 28 days of plant growth confirmed the Cd^2+^ retention levels (Fig 9). At the end of the experiment, the VC substrate had high amounts of bioavailable and exchangeable Cd^2+^. Plants grown in this material had low Cd^2+^ contents in their tissues, confirming that the Cd^2+^ in VC was not easily released and thus that VC has a strong Cd^2+^ retention capacity. The VCR substrate had a higher amount of exchangeable Cd^2+^ than bioavailable Cd^2+^ and supplied low amounts of Cd^2+^ for plant uptake. This finding suggests that the relatively small amount of bioavailable Cd^2+^ released by VCR either formed weak bonds with the VCR or was complexed at more superficial binding sites. A similar result was observed for biochar; plants grown in this material accumulated large amounts of Cd^2+^, indicating weak or superficial bonds between the biochar and Cd^2+^. A large amount of exchangeable Cd^2+^ remained in the biochar at the end of the experiment, suggesting that Cd^2+^ retention by biochar occurs primarily through electrostatic bonds or through bonds in the interior of the material. Similar results were observed for humin, which had the lowest exchangeable Cd^2+^ values of any of the tested materials at the end of the experiment; thus, the Cd^2+^ released by humin during its interaction with plants was probably adsorbed through weak chemical bonds.

In the present study, we evaluated natural materials that are commonly used for phytoremediation of soils contaminated by heavy metals. Biochar showed a high capacity for Cd^2+^ retention. However, when all binding sites are occupied, biochar makes large amounts of Cd^2+^ available to plants via interaction with roots, and this Cd^2+^ accumulates in plant tissues. This finding indicates that biochar should be used with care for phytoremediation. Other authors have expressed concerns regarding the use of biochar for phytoremediation based on studies conducted under other experimental conditions [60,61]. Humin is not recommended for phytoremediation because, in addition to possessing the lowest Cd^2+^ retention capacity, it easily made Cd^2+^ available to plants. In this study, VC and VCR showed the most promising results. VC had high Cd^2+^ retention and resulted in the lowest plant Cd^2+^ accumulation. In addition, shoot growth was increased by VC in the presence of retained Cd^2+^. VCR had both high Cd^2+^ retention and low Cd^2+^ bioavailability. The observed capacity of VCR for heavy metal retention is consistent with previous reports [30].

## Conclusion

The structural characteristics of the materials tested affect their Cd^2+^ retention and Cd^2+^ bioavailability and consequently determine the Cd^2+^ toxicity to rice plants. The results of this study have practical implications, indicating that rigorous monitoring should be employed when biochar is used for remediation of Cd^2+^-contaminated soils or to increase crop yields in soils with unknown degrees of heavy metal contamination. In addition, we conclude that humin is not effective for soil remediation due to its low Cd^2+^ retention and high capacity to release bioavailable Cd^2+^ to plants. In contrast, VC proved to be a promising material for the remediation of Cd^2+^-contaminated soils. However, because humic substances may form stable compounds with Cd^2+^, other environmental concerns that were not evaluated in the present study should be investigated (for example, solubility). The most promising material tested in this study was VCR. This is a novel result that has not been reported in previous studies.

## Supporting Information

S1 Fig Scanning electron microscopy of biochar used in this study. (TIF)

S2 Fig CP/MAS ^13^C-NMR spectra of the materials with and without levels of retained Cd^2+^. (TIF)

S3 Fig FTIR spectra of the tested materials with and without various levels of retained Cd^2+^. (TIF)

S1 Table Amount of type carbon (%) obtained by integrating areas in the CP/MAS ^13^C-NMR spectra with and without the retained metal. (DOCX)
