# Supplementary figures and images for: Assessment of the Use of Natural Materials for the Remediation of Cadmium Soil Contamination
Source: PLoS One. 2016 Jun 24;11(6):e0157547. doi: 10.1371/journal.pone.0157547 (PMC4920433; doi:10.1371/journal.pone.0157547)

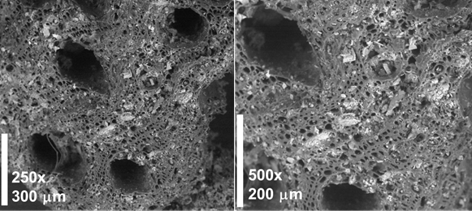

Supplement: S1 Fig — (TIF) [file pone.0157547.s001.tif]

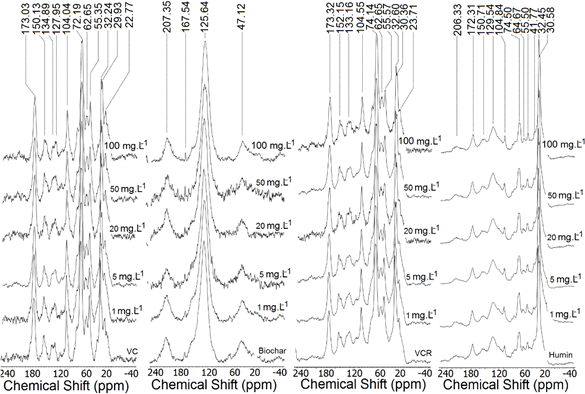

Supplement: S2 Fig — (TIF) [file pone.0157547.s002.tif]

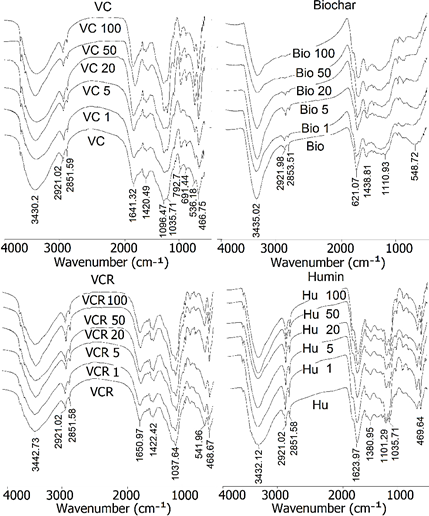

Supplement: S3 Fig — (TIF) [file pone.0157547.s003.tif]
