# Supplementary material for: Assessment of the Use of Natural Materials for the Remediation of Cadmium Soil Contamination
Source: PLoS One. 2016 Jun 24;11(6):e0157547. doi: 10.1371/journal.pone.0157547 (PMC4920433; doi:10.1371/journal.pone.0157547)
Supplement: S1 Table — (DOCX) [file pone.0157547.s004.docx]

| **Materials** | **0-46** | **46-59** | **59-91** | **91-110** | **110-142** | **142-156** | **156-186** | **186-230** | **Arom** | **Aliph** |
| --- | --- | --- | --- | --- | --- | --- | --- | --- | --- | --- |
|  | **CAlk-H,R** | **CAlk-O,N** | **CAlk-O** | **CAlk-di-O** | **CAr-H,R** | **CAr-O,N** | **CCOO-H,R** | **CC=O** |  |  |
| **Hu** | 39.29 | 7.14 | 15.18 | 4.46 | 19.64 | 5.36 | 7.14 | 1.79 | 25.00 | 75.00 |
| **Hu 1 mg.L^-1^** | 54.13 | 6.42 | 13.76 | 4.59 | 16.51 | 2.75 | 1.83 | 0.00 | 19.27 | 80.73 |
| **Hu 5 mg.L^-1^** | 38.39 | 7.14 | 16.07 | 4.46 | 19.64 | 5.36 | 7.14 | 1.79 | 25.00 | 75.00 |
| **Hu 20 mg.L^-1^** | 36.61 | 7.14 | 16.07 | 4.46 | 19.64 | 5.36 | 8.04 | 2.68 | 25.00 | 75.00 |
| **Hu 50 mg.L^-1^** | 28.85 | 7.69 | 17.31 | 4.81 | 22.12 | 6.73 | 9.62 | 2.88 | 28.85 | 71.15 |
| **Hu 100 mg.L^-1^** | 33.91 | 6.96 | 16.52 | 4.35 | 20.00 | 6.09 | 8.70 | 3.48 | 26.09 | 73.91 |
| **VCR** | 20.80 | 10.40 | 37.60 | 9.60 | 10.40 | 4.80 | 6.40 | 0.00 | 15.20 | 84.80 |
| **VCR 1 mg.L^-1^** | 19.82 | 9.01 | 37.84 | 9.91 | 9.91 | 5.41 | 8.11 | 0.00 | 15.32 | 84.68 |
| **VCR 5 mg.L^-1^** | 20.00 | 9.09 | 37.27 | 9.09 | 10.91 | 5.45 | 8.18 | 0.00 | 16.36 | 83.64 |
| **VCR 20 mg.L^-1^** | 19.82 | 9.91 | 36.04 | 9.91 | 10.81 | 4.50 | 9.01 | 0.00 | 15.32 | 84.68 |
| **VCR 50 mg.L^-1^** | 19.82 | 9.91 | 36.04 | 9.91 | 10.81 | 5.41 | 8.11 | 0.00 | 16.22 | 83.78 |
| **VCR 100 mg.L^-1^** | 19.82 | 9.01 | 36.04 | 10.81 | 10.81 | 5.41 | 8.11 | 0.00 | 16.22 | 83.78 |
| **VC** | 17.86 | 9.82 | 33.93 | 9.82 | 12.50 | 536 | 10.71 | 0.00 | 17.85 | 82.14 |
| **VC 1 mg.L^-1^** | 20.54 | 9.82 | 33.93 | 8.93 | 11.61 | 5.36 | 9.82 | 0.00 | 16.96 | 83.03 |
| **VC 5 mg.L^-1^** | 19.47 | 9.73 | 35.40 | 9.73 | 11.50 | 4.42 | 973 | 0.00 | 15.92 | 84.07 |
| **VC 20 mg.L^-1^** | 19.47 | 9.73 | 34.51 | 9.73 | 12.39 | 4.42 | 9.73 | 0.00 | 16.81 | 83.18 |
| **VC 50 mg.L^-1^** | 23.81 | 10.48 | 32.38 | 8.57 | 10.48 | 4.76 | 9.52 | 0.00 | 15.23 | 84.76 |
| **VC 100 mg.L^-1^** | 24.27 | 9.71 | 32,04 | 8.74 | 10.68 | 4.85 | 9.71 | 0.00 | 15.53 | 84.46 |
| **Biochar** | 10.57 | | **---** | 0.96 | 1.92 | 62.50 | 8.65 | 2.88 | 71.15 | 28.84 |
| **Bio1 mg.L^-1^** | 9.47 | | **---** | 0.00 | 1.05 | 67.36 | 11.57 | 5.26 | 78.94 | 21.05 |
| **Bio 5 mg.L^-1^** | 17.28 | | **---** | 3.70 | 7.40 | 62.96 | 9.87 | 6.17 | 72.83 | 27.16 |
| **Bio 20 mg.L^-1^** | 17.44 | | **---** | 2.32 | 4.65 | 70.93 | 8.13 | 2.32 | 79.06 | 20.93 |
| **Bio 50 mg.L^-1^** | 14.77 | | **---** | 3.40 | 5.68 | 72.72 | 6.81 | 1.13 | 79.54 | 20.45 |
| **Bio 100 mg.L^-1^** | 15.90 | | **---** | 2.27 | 5.68 | 70.45 | 7.95 | 2.27 | 78.40 | 21.59 |
